# Kinase insert domain receptor/vascular endothelial growth factor receptor 2 (KDR) genetic variation is associated with ovarian hyperstimulation syndrome

**DOI:** 10.1186/1477-7827-12-36

**Published:** 2014-05-09

**Authors:** Travis J O’Brien, Arthur F Harralson, Tuyen Tran, Ian Gindoff, Funda E Orkunoglu-Suer, David Frankfurter, Paul Gindoff

**Affiliations:** 1 Department of Pharmacology and Physiology, The George Washington University, Washington, DC, USA; 2 Department of Pharmacogenomics, Bernard J. Dunn School of Pharmacy, Shenandoah University, Ashburn, VA, USA; 3 Center for Neuroscience, Children’s National Medical Center, Washington, DC 20010, USA; 4 Quest Diagnostics, Athena Diagnostics, Worcester, MA 01605, USA; 5 Department of Obstetrics and Gynecology, The George Washington University, Washington, DC, USA

**Keywords:** Ovarian stimulation, Ovarian hyperstimulation syndrome, OHSS, KDR, VEGFR2, Polymorphism

## Abstract

**Background:**

The objective of this investigation was to determine if kinase insert domain/vascular endothelial growth factor receptor 2 (KDR/VEGFR2) genetic variation was associated with the development of ovarian hyperstimulation syndrome (OHSS) in patients undergoing controlled ovarian hyperstimulation (COH).

**Methods:**

This was a case–control study of 174 patients who underwent controlled ovarian stimulation. Patient blood samples were genotyped for single nucleotide polymorphisms (SNPs) spanning the *KDR* locus. OHSS development, clinical outcome variables, SNP and haplotype frequencies were compared between control (n = 155) and OHSS (n = 19) groups.

**Results:**

Patients who developed OHSS had significantly higher response markers (estradiol levels of the day of hCG administration, number of follicles developed, number of eggs retrieved) than control patients. When adjusted for age and self-identified race, the rs2305945 G/T genotype was associated (P = 0.027) with a decreased risk (OR = 0.30; 95% CI = 0.10, 0.93) of developing OHSS using an overdominant model. The rs2305945 G/T variant was also associated with decreased COH response (number of follicles, number of eggs retrieved) in an overdominant model. The rs2305948, rs1870378, rs2305945 (C-T-G) haplotype was associated with both decreased COH response and OHSS risk (unadjusted OR = 0.10; 95% CI = 0.01, 0.80, P = 0.031).

**Conclusions:**

The KDR receptor is believed to play a central role OHSS development and is a target for pharmacological prevention of OHSS. These results indicate that genetic variation in the *KDR* gene may impact individual risk of developing OHSS from COH. In addition, the rs2305948 SNP and C-T-G haplotype might serve as potential biomarkers for poor ovarian response to COH.

## Background

Controlled ovarian hyperstimulation (COH) has played a leading role in improving outcomes from *in vitro* fertilization (IVF). The backbone of COH pharmacotherapy involves the use of exogenous gonadotropins. While there are several clinical predictors [1-4] of ovarian responsiveness that aid in individualizing COH [5,6], interindividual variation in response to gonadotropin administration still exists. Additionally, patient risk of developing complications from COH is similarly unpredictable.

For the normal responder, COH is associated with some degree of risk for the iatrogenic complication ovarian hyperstimulation syndrome (OHSS). Potentially life threatening, OHSS leads to hospitalization in 1.9% of IVF cases [7]. Moderate to severe OHSS may be underestimated since many such cycles are frequently cancelled or result in the cryopreservation of all embryos [8]. Mild OHSS is relatively common with symptoms including abdominal bloating and mild weight gain. OHSS is characterized by cystic enlargement of the ovaries, increased vascular permeability (VP) and movement of fluid from the peritoneal vasculature into the third space. Symptoms and signs of severe OHSS include dyspnea, hemoconcentration, diminished renal perfusion and thromboembolism [9,10]. In cases of severe OHSS, myocardial infarction and/or stroke can lead to death [9,10]. Early onset OHSS occurs within 3–5 days after oocyte retrieval and is related to the hyper-response of the ovaries to COH followed by the use of hCG for oocyte maturation. Late onset OHSS appears 9–12 days after oocyte retrieval and results from COH and the endogenous hCG produced by a developing embryo [11]. Although rare, spontaneous cases of OHSS have been reported with the supra-physiologic hCG levels seen with multiple gestations or molar pregnancy [12]. Reports of familial spontaneous gestational OHSS suggest a genetic cause [13]. Regardless of severity, the development of OHSS is associated with significant physical, psychological and financial implications [14].

Standard practice emphasizes an avoidance approach in preventing OHSS (see below). There are several known risk factors for OHSS development that can assist in tailoring gonadotropin dosage [15-17]. In addition, markers for COH response (i.e. baseline anti-mullerian hormone (AMH) levels, estradiol levels, intermediate follicle number and FSH levels) may serve as early indicators allowing corrective measures to be taken to decrease severity of OHSS. Specific preventative strategies include coasting, avoiding the use of hCG, delaying/reducing hCG administration or cryopreserving all embryos [18]. Unfortunately, beyond avoiding hCG for oocyte maturation, most current, proactive measures are not completely effective in preventing OHSS.

The molecular etiology of OHSS is unclear. Elevated serum estradiol [14], cytokine and interleukin levels have all been detected in the peritoneal fluid of women with OHSS [19,20]. Moreover, roles for renin-angiotensin, prolactin and prostaglandins in the increased VP observed during OHSS have also been proposed [21]. The most important VP factor in the ovaries is vascular endothelial growth factor (VEGF). In rats, VEGF mRNA levels and VP increased following gonadotropin stimulation [22] which was reversed by VEGF antiserum [23]. In humans, hCG administration increased VEGF expression in granulosa-lutein cells [24] and VEGF blood levels predicted the development of OHSS and its severity [25]. In addition, a single nucleotide polymorphism in the *VEGF* gene has been associated with increased OHSS risk [26]. Consequently, the prevailing model for OHSS development involves aberrant VEGF signaling as a key factor driving increased VP [23,27].

In the ovaries, VEGF-mediated VP, at least in part, is mediated by the kinase insert domain/vascular endothelial growth factor receptor 2 (KDR/VEGFR-2/Flk-1) signaling mechanisms [28-33]. In support of this, inhibition of both VEGF [34,35] and KDR [22,31,32,35] has been shown to ameliorate VP development in models of OHSS. In addition, dopamine [36] and [37] dopamine receptor agonists [20,27,38-40] are known inhibit KDR function [36,41] and show promise as both preventative and therapeutic options for OHSS [39,42-45].

Despite much research on the topic, very few predictive genetic biomarkers exist for COH outcome [46]. Genetic variation in *FSHR*[47], *CYP19A1*[47], *BMP15*[48], VEGF [26] and *LHCGR*[49] have all been associated with high response to COH and/or OHSS. *KDR* has been implicated in the etiology of OHSS and also serves as a target for pharmacotherapy [20,38,40,45,50]. However, no information is available on the association of KDR genetic variability and OHSS risk. As a result, the focus of this investigation was to evaluate the role of KDR polymorphisms in the development of OHSS.

## Methods

This study was approved by George Washington University Institutional Review Board. Patients’ and written informed consent was obtained prior to enrollment of patients (2010–2011). All IVF patients (>18 years of age) who were treated at the GW Fertility and IVF Center with injectable gonadotropins were invited to participate. All patients were evaluated for ovarian reserve testing, semen analysis (male partner), uterine cavity study and thyroid screening. Controlled ovarian stimulation protocols were as previously described [49]. After initial follicular monitoring (serum estradiol and transvaginal ultrasound assessments), FSH dosing was titrated based upon the ovarian response. hCG trigger was withheld for E2 levels over 4000 pg/ml thus minimizing risk for OHSS. Both control and OHSS groups had similar risk factors including those identified at time of hCG trigger. Ovarian hyperstimulation syndrome was defined clinically based on established criteria [51,52]. For each patient the following clinical endpoints were recorded: estradiol level on day of hCG injection, number of ovarian follicles on day of hCG, number of follicles/follicles >16 mm), number of eggs retrieved and the incidence of OHSS.

For each patient, blood (5 mL) was collected and DNA was extracted using a QiaCube automated instrument with the QIAamp DNA Blood Mini Kit (Qiagen, Valencia, CA). KDR/VEGFR-2 SNPs rs3025035, rs2305948, rs2219471, rs1870378, rs2305945 and rs1870377 were genotyped using Real-Time PCR (*Taq*Man®). PCR was performed with a reaction volume of 10 μl, including 4.75 μl of *Taq*Man® Universal PCR Master Mix, 0.5 μl of 20X DME Genotyping Assay Mix, 3.75 μl of DEPC H_2_O, and 1.0 μl of genomic DNA. The PCR cycling conditions were as follows: 1 cycle of 50°C for 2 minutes, followed by 1 cycle of 95°C for 10 minutes and 50 cycles of 92°C for 15 seconds and 60°C for 90 seconds. Appropriate negative controls were included with each run. Allelic discrimination was carried out by measuring fluorescence intensity using an ABI 7300 Real Time PCR System (Applied Biosystems) and SDS software Version 1.3 (Applied Biosystems). PCR/sequencing primers are shown in Table 1. The genotype calls for each SNP were verified in subsets of samples by DNA sequencing as previously described [49] using PCR primers in Table 1.

**Table 1:** Primer sequences for DNA sequencing

**SNP**	**Forward (5′ to 3′)**	**Reverse (5′ to 3′)**
rs2219471	TCCACAGGGATTGCTCCAAC	ATATTTGGCCCCGTGGAGTG
rs3025035	CAGGGGTCCTTGGGAAAGAT	AGAACAGGCCCTACCCTTCT
rs2305945	GTGGGTACTAAGCTATGTAATTCCC	CCACACAGAGCTTGTGGTTTA
rs2305948	TTTCCAAGACCATAGCTTACCAT	CAGCATCAGCATAAGAAACTTGTA
rs1870377	TGGTACTGCTAAAAGTCAATGG	GGCTGCGTTGGAAGTTATTT
rs1870378	CACTACGGCCTCAAGAGAGAAG	CTGGGTTCCCAAATGTTATGCG

Comparisons between OHSS and control samples for each of the clinical variables were conducted using SPSS (IBM, Armonk, NY). Due to the skewed nature of the data an independent samples Mann–Whitney *U* test was used for all comparisons. SNPs spanning the KDR locus were selected using Haploview (version 4.2, r^2^ > 0.75) [53] (CEU population) [54]. Analyses for Hardy-Weinberg equilibrium, linkage disequilibrium (LD) and unadjusted odds ratios for OHSS risk were conducted using SNPSTATS [55] and HAPSTAT [56].

## Results

We obtained genotypic and clinical information for 174 patients who underwent IVF. We have previously reported on the association of *LHCGR* rs4073366 C allele carrier status with OHSS (n = 172) risk in this patient population [49]. However, we did not perform statistical comparisons of the control and OHSS populations’ clinical characteristics. OHSS patients (n = 19) had significantly greater COH response markers than control patients (n = 155). Specifically, estradiol (E_2_) levels (median; 25%, 75% percentile) on the day of hCG administration [Controls: 1735.0 pg/mL (1031.0-2280.0); OHSS: 2606.0 pg/mL (1996.0-3471.0)], number of follicles (median; 25%, 75% percentile) generated [Controls: 2.0 (1.0-4.0); OHSS: 5.0 (3.0-10.0)] and number of eggs (median; 25%, 75% percentile) retrieved [Controls: 8.0 (4.0-13.0); OHSS: 18.0 (13.0-26.0)] were all significantly (P < 0.001) higher in OHSS patients. In addition, OHSS occurred to a greater extent in patients of self-identified Caucasian ethnicity versus Black, non-Hispanic or Asian/Pacific Islander [49].

Six SNPs in the *KDR* gene were investigated for association with OHSS. SNPs were selected based on pair-wise linkage disequilibrium analysis using HapMap [57] data (CEU population) in the *KDR* for the prediction of haplotype blocks. The 6 SNPs (rs3025035, rs2305948, rs2219471, rs1870378, rs2305945, rs1870377) spanning the *KDR* gene were selected and genotyped in both control and OHSS patients. SNPs that were not in Hardy Weinberg equilibrium (rs3025035, rs2219471, rs1870377) were omitted from further analysis. The remaining SNPs rs2305948, rs1870378 and rs2305945 were not in linkage disequilibrium (See Additional file 1: Table S1) and existed in 3 separate predicted haplotype blocks (data not shown). Specifically, tagged SNPs rs1870378 and rs2305945 included rs2219471 and rs6838752 (covering ~5 kb) and rs3828550 and rs13109660 (covering ~5 kb), respectively.

For rs2305948, C/C, C/T and T/T variants occurred at frequencies of 0.75, 0.23 and 0.02, respectively in the entire patient population (Table 2). The observed frequencies for rs1870378 C/C, C/T and T/T genotypes were 0.55, 0.37 and 0.07 of all patients in the study. The rs2305945 G/G, G/T and T/T variants were found at frequencies of 0.40, 0.45 and 0.14 in the total population as well. Although there were differences in the frequencies of SNPs in OHSS case versus control patients, none reached statistical significance (alpha of 0.05).

**Table 2:** SNP frequencies in controls and OHSS cases

**Variant**	**Frequency**		
	**Total (n = 174)**	**Controls (n - 155)**	**OHSS Cases (n = 19)**
*rs2305948*			
CC	131 (0.75)	116 (0.75)	15 (0.79)
CT	40 (0.23)	36 (0.23)	4 (0.21)
TT	3 (0.02)	3 (0.02)	0
*rs1870378*			
CC	96 (0.55)	83 (0.54)	13 (0.68)
CT	65 (0.37)	60 (0.39)	5 (0.26)
TT	13 (0.07)	12 (0.08)	1 (0.05)
*rs2305945*			
GG	70 (0.40)	61 (0.39)	9 (0.47)
GT	79 (0.45)	74 (0.48)	5 (0.26)
TT	25 (0.14)	20 (0.13)	5 (0.26)

None of the individual SNPs were independent predictors (unadjusted) of OHSS risk (data not shown). In an overdominant model, rs23205945 was associated (P = 0.031) with decreased OHSS risk (OR = 0.30; 95% CI = 0.10, 0.93) when corrected for age and self-identified race (Table 3). When adjusted for age (P = 0.017), race (P = 0.017) or both age and race (P = 0.013), the rs2305945 G/T genotype was significantly associated with fewer follicles generated by COH (See Additional file 2: Table S2). In addition, the rs2305945 G/T genotype was marginally associated (P = 0.046) with a fewer number of eggs retrieved in an overdominant model only after adjustment for self-identified race (See Additional file 3: Table S3).

**Table 3:** rs2305945 association with OHSS (n = 174, Adjusted for Age and Race)

**Model**	**Genotype**	**Controls**	**OHSS**	**OR (95% CI)**	**P-value**
Codominant	G/G	61 (39.4%)	9 (47.4%)	1	0.064
	G/T	74 (47.7%)	5 (26.3%)	0.35 (0.10, 1.19)	
	T/T	20 (12.9%)	5 (26.3%)	1.70 (0.46, 6.34)	
Dominant	G/G	61 (39.4%)	9 (47.4%)	1	0.320
	G/T-T/T	94 (60.6%)	10 (52.6%)	0.59 (0.21, 1.65)	
Recessive	G/G-G/T	135 (87.1%)	14 (73.7%)	1	0.110
	T/T	20 (12.9%)	5 (26.3%)	2.79 (0.82, 9.47)	
Overdominant	G/G-T/T	81 (52.3%)	14 (73.7%)	1	0.027
	G/T	74 (47.7%)	5 (26.3%)	0.30 (0.10, 0.93)	

A significant difference in haplotype distribution between OHSS cases and control patients (P = 0.033) was observed. Interestingly, one (rs2305948, rs1870378, rs2305945; T-T-T) of the eight possible haplotypes was not observed in either the OHSS case or control populations (Table 4). Two haplotypes (T-C-G, T-C-T) were not detected in the OHSS population, but also occurred at low frequencies (<5.0%) in the control population as well. All missing haplotypes were not included in the haplotype-based logistic regression analysis. The rs2305948, rs1870378, rs2305945 (C-T-G) haplotype (unadjusted) was found to be moderately protective (P = 0.031) for OHSS risk (OR = 0.10; 95% CI = 0.01, 0.80) (Table 5). When adjusted for age (P = 0.020), race (P = 0.023) or age/race (P = 0.011), the C-T-G haplotype was significantly associated with decreased OHSS development (Table 6). Additionally, COH response variables number of follicles >16 mm and eggs retrieved were significantly lower in the C-T-G haplotype (See Additional file 4: Table S4 and Additional file 5: Table S5). Only one other haplotype (C-C-T) was significantly associated with an endpoint (fewer follicles > 16 mm) (See Additional file 6: Table S6).

**Table 4:** Haplotype estimation (n = 174)

**Haplotype**			**Frequency**		
**rs2305948**	**rs1870378**	**rs2305945**	**Total**	**Control**	**OHSS Cases**
C	C	G	0.352	0.334	0.474
C	C	T	0.327	0.329	0.342
C	T	G	0.164	0.185	0.026
T	T	G	0.073	0.069	0.105
T	C	G	0.041	0.044	--
C	T	T	0.025	0.017	0.053
T	C	T	0.019	0.022	--
T	T	T	--	--	--

**Table 5:** Haplotype frequencies estimation and association with OHSS (n = 174)

**Haplotype**	**rs2305948**	**rs1870378**	**rs2305945**	**OR (95% CI)**	**P-value**
1	C	C	G	1	---
2	C	C	T	0.72 (0.32, 1.59)	0.42
3	C	T	G	0.10 (0.01, 0.80)	0.031
4	T	T	G	1.15 (0.34, 3.87)	0.82
5	T	C	G	--	--
6	C	T	T	2.72 (0.35, 21.30)	0.34
7	T	C	T	--	--

Global haplotype association p-value: 0.033.

**Table 6:** **Haplotype (CTG) assoc**i**ation with OHSS risk**

**Haplotype**	**OR**	**95% CI**	**P-value**
*rs2305948 (C), rs1870378 (T), rs2305945 (G)*			
**Unadjusted**			
	0.10	0.01, 0.80	0.031
**Adjusted**			
Age	0.08	0.01, 0.66	0.020
Race	0.08	0.01, 0.69	0.023
Age, Race	0.04	0.00, 0.46	0.011

## Discussion

The aim of this investigation was to determine whether *KDR* genetic variation was associated with OHSS risk in COH patients. We found a novel association between the *KDR* rs2305948, rs1870378, rs2305945 C-T-G haplotype and reduced risk of developing OHSS. Patients with this haplotype also exhibited decreased ovarian response to COH (i.e. number of follicles >16 mm, eggs retrieved). In addition, the rs2305945 G/T variant was similarly associated with decreased response to COH and lower risk of hyperstimulation. These findings are the first to suggest that *KDR* polymorphisms might serve as predictive genetic biomarkers for ovarian response to COH.

A central component of the pathophysiology of iatrogenic OHSS is increased ovarian VP during COH. The molecular mechanism for increased VP is thought to involve aberrant VEGF signaling [58]. Serum VEGF levels are elevated in OHSS and predictive for OHSS risk [59]. In addition, a *VEGFA* polymorphism has been as recently identified as a risk allele for OHSS [26]. VEGF-mediated VP is thought to act through KDR-dependent mechanisms and dopamine/dopamine receptor agonists [27,36], which purportedly inhibit KDR function, have shown promise as therapies for OHSS [27,39,42-44]. Interestingly, we observed a moderate association between the (rs2305948/rs1870378/rs2305945) C-T-G haplotype and lower OHSS risk. Given that the pathophysiology of OHSS involves increased VP, these results suggest that C-T-G haplotype could potentially result in a KDR receptor with decreased function.

The C-T-G haplotype included two intronic SNPs: rs1870378 (in intron 15) and rs2305945 (intron 12). Neither of these polymorphisms have been associated with disease risk or clinical outcomes. In contrast, the rs2305948 (G > A) variant is a nonsynonymous SNP located in exon 7 that results in an amino acid change from valine to isoleucine (codon 297). It resides in the NH_2_-terminal portion of the receptor located in the extracellular, ligand-binding domain. *In vitro* evidence suggests that rs2305948 (G > A) variant decreases KDR binding to VEGF [60]. Clinically, rs2305948 has been associated with increased risk of coronary artery disease [60], intracerebral hemorrhage and stroke recurrence [61]. In addition, the rs2305948 T allele exists in a haplotype with rs10020464 and rs7692791 that was moderately associated with a lower risk of developing neovascular age-related macular degeneration [62]. However, we found that the rs2305948 C allele in the *C*-T-G haplotype was associated with decreased OHSS risk. Therefore, the exact role, if any, which the rs2305948 C allele plays in the apparent protection from OHSS provided by the C-T-G haplotype, requires further investigation.

We found only one polymorphism to be moderately associated with OHSS development when corrected for co-variates (age, race) known to be independent predictors of OHSS risk [8,49]. The rs2305945 G/T genotype was associated with a reduced likelihood of OHSS (OR = 0.30; 95% CI = 0.10, 0.93) in an overdominant model. This SNP resides within intron 12 located ~153 bp downstream of exon 12. It is intriguing that the rs2305945 genotype and the C-*T*-G haplotype were associated with both decreased ovarian response and lower risk of OHSS. We postulate that rs2305945 SNP could impact *KDR* mRNA processing and/or stability leading to decreased downstream signaling. However, the precise mechanism by which the G/T heterozygote impacts KDR function requires additional mechanistic studies.

The majority of poor responders to COH suffer from reduced ovarian reserve [63]. While this study has identified potential protective genetic biomarkers for OHSS, the results are also potentially applicable to identifying patients with diminished ovarian reserve (DOR). To date, there are very few genetic biomarkers for DOR [64-67]. We found that the rs2305945 SNP and the C-T-G haplotype were both associated with diminished ovarian response to COH. It would be interesting to specifically investigate the frequency of these variants in COH poor responders and/or patients with DOR. As a result, our results offer promise that *KDR* polymorphisms might also serve as novel, predictive biomarkers for DOR in COH patients.

The KDR receptor plays an integral role in ovarian VP and has shown promise as a target for pharmacologic intervention to prevent OHSS. However, there is no information available on the impact of *KDR* polymorphisms on patient risk of developing OHSS during COH. The results from this preliminary study indicate that *KDR* polymorphisms are potential predictive biomarkers for OHSS development. We believe this is the first study to link *KDR* polymorphisms with OHSS risk and decreased ovarian response to COH. A limitation of the study was the small number of OHSS cases available for analysis. The significance of these findings requires validation in a larger, separate population of patients. Given that the variants identified in this study have individually small effect sizes, future work is aimed at uncovering other risk alleles in *KDR* and other genes implicated in ovarian angiogenesis and VP.

## Conclusions

The KDR receptor plays a central role in VEGF-mediated vascular permeability in OHSS and represents a potential target for pharmacologic intervention of OHSS. These results indicate that genetic variation in the *KDR* gene may impact individual risk for developing OHSS from COH. In addition, these results suggest that the rs2305948 variant and C-T-G haplotype may serve as potential biomarkers for diminished response to COH.

## Abbreviations

COH: Controlled ovarian hyperstimulation; OHSS: Ovarian hyperstimulation syndrome; SNP: Single nucleotide polymorphism; VEGFR2: Vascular endothelial growth factor receptor 2; KDR: Kinase insert domain receptor.

## Competing interests

The authors declare that they have no competing interests associated with this work.

## Authors’ contributions

TO: Conceived of the study, participated in its design, carried out molecular analyses, assisted in data analysis and drafted the manuscript. PG: Conceived of the study, participated in its design, recruited participants, assisted with drafting the manuscript. AH: Participated in study design, assisted in data analysis and drafted the manuscript. FOS: carried out molecular analysis and assisted in data analysis. DF: Conceived of the study, participated in its design, recruited participants, assisted with drafting the manuscript. TT: carried out molecular analyses and assisted in data analysis. IG: carried out molecular analyses and assisted in data analysis All authors read and approved the final manuscript.

## Supplementary Material

Additional file 1: Table S1 Linkage Disequilibrium Analysis (D’ statistic). Click here for file

Additional file 2: Table S2 rs2305945 association with number of follicles in overdominant model (n = 174). Click here for file

Additional file 3: Table S3 rs2305945 association with number of eggs retrieved (n = 174). Click here for file

Additional file 4: Table S4 Haplotype (CTG) association with large (>16 mm) follicles. Click here for file

Additional file 5: Table S5 Haplotype (CTG) association with number of eggs retrieved. Click here for file

Additional file 6: Table S6 Haplotype (CCT) association with large (>16 mm) follicles. Click here for file
