# Supplementary material for: Kinase insert domain receptor/vascular endothelial growth factor receptor 2 (KDR) genetic variation is associated with ovarian hyperstimulation syndrome
Source: Reprod Biol Endocrinol. 2014 May 9;12:36. doi: 10.1186/1477-7827-12-36 (PMC4024119; doi:10.1186/1477-7827-12-36)
Supplement: Additional file 1: Table S1 — Linkage Disequilibrium Analysis (D’ statistic). [file 1477-7827-12-36-S1.docx]

# Additional files

**Additional file 1, Supplemental Table S1**

**Linkage Disequilibrium Analysis^*^**

| **SNP** | **rs2305948** | **rs1870378** | **rs2305945** |
| --- | --- | --- | --- |
| rs2305948 | -- | 0.382 | 0.575 |
| rs1870378 | -- | -- | 0.745 |
| rs2305945 | -- | -- | -- |

**^*^** D' statistic
