# Supplementary material for: Kinase insert domain receptor/vascular endothelial growth factor receptor 2 (KDR) genetic variation is associated with ovarian hyperstimulation syndrome
Source: Reprod Biol Endocrinol. 2014 May 9;12:36. doi: 10.1186/1477-7827-12-36 (PMC4024119; doi:10.1186/1477-7827-12-36)
Supplement: Additional file 2: Table S2 — rs2305945 association with number of follicles in overdominant model (n = 174). [file 1477-7827-12-36-S2.docx]

**Additional Files**

**Additional file 2, Supplemental Table S2**

**rs2305945 association with number of follicles in overdominant model (n=174)**

| **Co-variate** | **Genotype** | **N** | **Response Mean (SE)** | **Difference (95% CI)** | **P-value** |
| --- | --- | --- | --- | --- | --- |
| Unadjusted | G/G-T/T | 94 | 11.87 (0.73) | 0 | 0.022 |
|  | G/T | 76 | 9.58 (0.64) | -2.29 (-4.24, -0.34) |  |
| Age | G/G-T/T | 94 | 11.87 (0.73) | 0 | 0.017 |
|  | G/T | 76 | 9.58 (0.64) | -2.23 (-4.4, -0.41) |  |
| Race | G/G-T/T | 94 | 11.87 (0.73) | 1 | 0.017 |
|  | G/T | 76 | 9.58 (0.64) | -2.39 (-4.34, -0.45) |  |
| Age and Race | G/G-T/T | 94 | 11.87 (0.73) | 1 | 0.013 |
|  | G/T | 76 | 9.58 (0.64) | -2.33 (-4.14, -0.52) |  |
