# Supplementary material for: Kinase insert domain receptor/vascular endothelial growth factor receptor 2 (KDR) genetic variation is associated with ovarian hyperstimulation syndrome
Source: Reprod Biol Endocrinol. 2014 May 9;12:36. doi: 10.1186/1477-7827-12-36 (PMC4024119; doi:10.1186/1477-7827-12-36)
Supplement: Additional file 3: Table S3 — rs2305945 association with number of eggs retrieved (n = 174). [file 1477-7827-12-36-S3.docx]

**Additional Files**

**Additional file 3, Supplemental Table S3**

rs2305945 association with number of eggs retrieved (n=174)

| **Model** | **Genotype** | **N** | **Response Mean (SE)** | **Difference (95% CI)** | | **P-value** | |  |
| --- | --- | --- | --- | --- | --- | --- | --- | --- |
| Codominant | G/G | 68 | 11.84 (1.09) | | 0 | | 0.103 | |
|  | G/T | 73 | 9.52 (0.7) | | -2.51 (-5.0, -0.03) | |  |  |
|  | T/T | 25 | 11.44 (1.49) | | -0.55 (-4.01, -2.91) | |  |  |
| Dominant | G/G | 68 | 11.84 (1.09) | | 0 | | 0.092 | |
|  | G/T-T/T | 98 | 10.01 (0.65) | | -2.01 (-4.35, -0.32) | |  |  |
| Recessive | G/G-G/T | 141 | 10.64 (0.65) | | 0 | | 0.650 | |
|  | T/T | 25 | 11.44 (1.49) | | 0.75 (-2.49, -3.99) | |  |  |
| Overdominant | G/G-T/T | 93 | 11.73 (0.89) | | 0 | | 0.046 | |
|  | G/T | 73 | 9.52 (0.70) | | -2.37 (-4.67, -0.06) | |  |  |
