# Supplementary material for: Kinase insert domain receptor/vascular endothelial growth factor receptor 2 (KDR) genetic variation is associated with ovarian hyperstimulation syndrome
Source: Reprod Biol Endocrinol. 2014 May 9;12:36. doi: 10.1186/1477-7827-12-36 (PMC4024119; doi:10.1186/1477-7827-12-36)
Supplement: Additional file 4: Table S4 — Haplotype (CTG) association with large (>16 mm) follicles. [file 1477-7827-12-36-S4.docx]

**Additional Files**

**Additional file 4, Supplemental Table S4**

Haplotype (CTG) association with large (>16 mm) follicles

| **Haplotype** | **Response Mean (SE)** | **95% C.I.** | **P-value** |
| --- | --- | --- | --- |
| *rs2305948 (C), rs1870378 (T), rs2305945 (G)* |  |  |  |
| **Unadjusted** |  |  |  |
|  | -0.69 | -1.95, -0.58 | 0.290 |
| **Adjusted** |  |  |  |
| Age | -1.07 | -2.10, -0.05 | 0.041 |
| Race | -0.88 | -2.24, -0.48 | 0.210 |
| Age, Race | -1.18 | -2.13, -0.23 | 0.016 |
