# Supplementary material for: Kinase insert domain receptor/vascular endothelial growth factor receptor 2 (KDR) genetic variation is associated with ovarian hyperstimulation syndrome
Source: Reprod Biol Endocrinol. 2014 May 9;12:36. doi: 10.1186/1477-7827-12-36 (PMC4024119; doi:10.1186/1477-7827-12-36)
Supplement: Additional file 5: Table S5 — Haplotype (CTG) association with number of eggs retrieved. [file 1477-7827-12-36-S5.docx]

**Additional Files**

**Additional file 5, Supplemental Table S5**

Haplotype (CTG) association with number of eggs retrieved

| **Haplotype** | **Response Mean (SE)** | **95% C.I.** | **P-value** |
| --- | --- | --- | --- |
| *rs2305948 (C), rs1870378 (T), rs2305945 (G)* |  |  |  |
| **Unadjusted** |  |  |  |
|  | -2.34 | -4.75, -0.07 | 0.059 |
| **Adjusted** |  |  |  |
| Age | -2.48 | -4.76, -0.20 | 0.034 |
