# Supplementary material for: Kinase insert domain receptor/vascular endothelial growth factor receptor 2 (KDR) genetic variation is associated with ovarian hyperstimulation syndrome
Source: Reprod Biol Endocrinol. 2014 May 9;12:36. doi: 10.1186/1477-7827-12-36 (PMC4024119; doi:10.1186/1477-7827-12-36)
Supplement: Additional file 6: Table S6 — Haplotype (CCT) association with large (>16 mm) follicles. [file 1477-7827-12-36-S6.docx]

**Additional Files**

**Additional file 6, Supplemental Table S6**

Haplotype (CCT) association with large (>16 mm) follicles

| **Haplotype** | **Response Mean (SE)** | **95% C.I.** | **P-value** |
| --- | --- | --- | --- |
| *rs2305948 (C), rs1870378 (C), rs2305945 (T)* |  |  |  |
| **Unadjusted** |  |  |  |
|  | -0.58 | -1.45, -0.29 | 0.190 |
| **Adjusted** |  |  |  |
| Age | -0.96 | -1.90, -0.02 | 0.046 |
| Race | -0.70 | -1.71, -0.32 | 0.180 |
| Age, Race | -1.05 | -1.83, -0.26 | 0.010 |
